# The impact of staged rehabilitation guidance based on symptom management theory on functional rehabilitation and quality of life of elderly patients undergoing hip replacement surgery

**DOI:** 10.12669/pjms.41.4.11693

**Published:** 2025-04

**Authors:** Yunwei Xu, Lvtian Chen

**Affiliations:** 1 Yunwei Xu, Department of Joint and Limb Surgery, The Affiliated Yangming Hospital of Ningbo University, Yuyao, Zhejiang Province 315400, P.R. China; 2 Lvtian Chen, Department of Joint and Limb Surgery, The Affiliated Yangming Hospital of Ningbo University, Yuyao, Zhejiang Province 315400, P.R. China

**Keywords:** Hip arthroplasty, Hip functional, Quality of life, Symptom management theory, Staged rehabilitation guidance

## Abstract

**Objective:**

Physical rehabilitation after hip arthroplasty (HA) is crucial for improving patients’ functional outcomes and quality of life (QOL). This study aimed to explore the impact of staged rehabilitation guidance based on symptom management theory (SMT) on functional rehabilitation and QOL in elderly patients undergoing HA.

**Methods:**

This retrospective case-control analysis included a cohort of 60 patients who received SMT-based staged rehabilitation guidance between January 2023 to April 2024 at The Affiliated Yangming Hospital of Ningbo University. Patients of the SMT group were matched 1:1 with patients who received routine care (the control group). The recovery of hip joint function, motor function, QOL, and self-care ability after intervention was compared.

**Results:**

After four and 12 weeks of intervention, the Harris hip score (HHS), functional independence measure (FIM) score, 6-minute walk test (6MWT), Berg balance scale (BBS) score, QLQ-C30, and Barthel index (BI) score of the SMT group were significantly higher, while the standing to walking time was significantly lower than that of the control group (P<0.05).

**Conclusions:**

The staged rehabilitation guidance based on SMT principles has potentially positive effects on the hip joint function, motor function, QOL, and self-care ability of elderly patients undergoing HA.

## INTRODUCTION

Hip arthroplasty (HA) is commonly used in treating elderly patients with hip fractures, femoral head necrosis, and hip joint degenerative diseases; it can effectively improve patient function and reduce the occurrence of complications.^1^–^3^ However, clinical findings show that about 25%～75% of elderly patients with HA cannot return to normal activities of daily living within one year after surgery,^2^–^4^ and the incidence of chronic pain after HA is about 30%,^4^,^5^ which makes improving functional rehabilitation crucial in this population.^3^–^5^

However, current clinical measures for rehabilitation after HA in the elderly are primarily based on the common characteristics of the disease and often fail to achieve the desired effect.^6^ A recent randomized controlled trial reported developing and evaluating a staged rehabilitation program in patients after knee arthroplasty.^7^ Guidance was provided throughout the rehabilitation process, with the exercise intensity progressing from low to high and the length of rehabilitation training from short to long, aiming to improve functional recovery and avoid excessive fatigue. However, the efficacy of this training method in the rehabilitation of HA patients is unclear.

SMT is a multidimensional and dynamic symptom management strategy that can provide high-quality nursing services for patients with common problems and individual needs and ensure a healthy outcome.^8^,^9^ At present, SMT-based nursing has been widely used to reduce the symptom experience of cancer patients, reduce complications, and improve the QOL.^9^

This study aimed to retrospectively analyze the clinical efficacy of the staged rehabilitation program based on the principles of SMT in elderly patients who underwent HA. Our results may contribute to developing more efficient rehabilitation methods for elderly patients after hip replacement.

## METHODS

**Fig.1:**
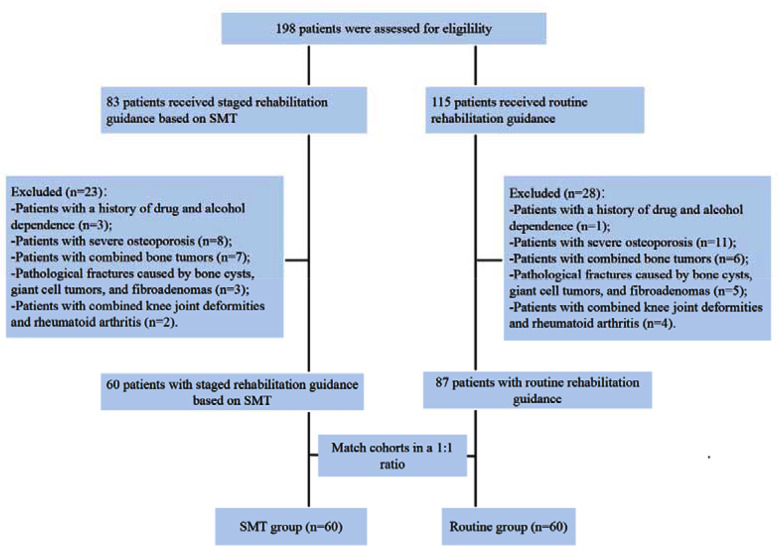
Participant screening flow chart; SMT: symptom management theory.

Clinical records of 120 elderly patients who underwent HA in The Affiliated Yangming Hospital of Ningbo University from January 2023 to April 2024 were retrospectively analyzed. Sixty patients received staged rehabilitation guidance based on SMT (SMT group), and a matched cohort of 60 patients received routine rehabilitation guidance (control group). The selection process is shown in Fig.1.

### Ethical Approval:

It was approved by the ethics committee of Yuyao People’s Hospital approved this study with the number 2024-10-001, Dated: Oct-17-2024.

### Inclusion criteria:

- Underwent HA.
- Age ≥ 60 years old.
- Unilateral onset.
- The clinical data were complete.

### Exclusion criteria:

- History of drug and alcohol dependence.
- Coagulation dysfunction.
- Severe osteoporosis.
- Osteoma.
- Pathological fracture caused by a bone cyst, giant cell tumor of bone, osteofibroma, etc.
- Patients with knee deformity and rheumatoid arthritis.

### Routine rehabilitation guidance:

One day after the surgery, ankle pump function exercise and upper limb joint activity training were initiated after ensuring the patient’s vital signs were stable. At one to three days after the surgery, patients were guided to carry out straight leg raising, leg spreading, hip raising, and muscle group training around the affected part while considering the patient’s tolerance. Five to seven days after the surgery, patients were instructed to carry out bedside standing and sitting exercises and walking aids-assisted walking exercises. Two weeks after the operation, the patients were instructed to carry out balance exercises, including standing on one leg, standing with eyes closed, etc. Hip and leg muscle strength exercises were carried out three to six weeks after the operation. Seven to 12 weeks after the operation, patients were guided to carry out daily-life activities such as washing, bathing, dressing independently, and going up and down stairs.

### Staged rehabilitation guidance based on SMT:

One day after the operation, after the patient’s vital signs were stable, they were instructed to lie flat on the bed and perform lying down limb exercises, ankle joint rotation, arm direction change, arm-opening, eye-opening and eye-closing exercises according to the instructions of the staff. Patients were asked to talk about their subjective feelings and report any discomfort.

Two days after the operation, the head of the bed was raised by about 30 °. The intensity of leg training, hip lifting, leg lifting, and other exercises was increased according to patients’ specific conditions. Patients were encouraged and assisted in carrying out exercises such as flexion, extension, and movement of affected limbs. The elevation angle of the head of the bed was further increased based on the patient’s tolerance and rehabilitation progress. Seven days after the operation, the head of the bed was raised to 90 °, and close attention was paid to whether the patient could bear such an elevation angle. In cases of discomfort, anxiety, and other manifestations, the training intensity was reduced, and the bed head elevation angle was adjusted appropriately. The angle was increased when patients were comfortable with the elevation of the head. Patients were assisted in carrying out bedside sitting and standing exercises, five minutes/time, 10 times/day, with an interval of one minute.

One week after surgery/at discharge, the rehabilitation physician and the attending physician jointly assessed the patient’s joint recovery, pain degree, and muscle strength and timely adjusted the rehabilitation plan. The responsibility of nurse was to educate patients and their families on the necessity of standardized rehabilitation training. For patients with negative emotions, measures were taken to relieve tension and anxiety in a timely manner.

Two weeks after the operation, according to the established rehabilitation guidance path, patients were encouraged to carry out standardized training and strengthen dietary and drug regimens. The rehabilitation training content was continuously explained and updated according to the patient’s rehabilitation status. Patients were guided to carry out basic daily living ability exercises, such as sitting and standing position changes, independent use of the toilet, independent dressing, wearing shoes (slippers), and other actions that do not require significant hip flexion and extension.

After patients successfully adapted to the basic daily living routine and did not report significant pain, they were encouraged to practice actions that require a certain range of motion of the hip joint and limb balance, such as wearing sports shoes, bathing, wearing pantyhose, and picking up things on the ground, as well as walking exercises. During the walking exercises, the patients were instructed to keep upright, look forward, raise the toe when starting, and follow the ground when landing. When landing, patients were instructed to follow the walking mark to control the step length and guided to adjust the step frequency according to the instructions.

Three to four weeks after the operation, motor function exercises were carried out, including going up and down stairs and crossing obstacles. Going up and down stairs was mainly carried out from one-stage discontinuous training, and the training ground was a single step with a height of about 15 cm (about 12 steps). During training, crutches could be used as an aid. When stepping onto the stage, patients were instructed to start with an uninjured limb, followed by crutches and then the affected limb. When stepping down the steps, patients were instructed to get the crutches down first and then use the crutches and the affected limbs as support to lower the healthy limbs.

Patients were instructed to perform three rounds of exercises/day, gradually increasing to 5-7 rounds/day according to the rehabilitation progress and tolerance. Eventually, patients were encouraged to carry out continuous up and down stairs exercises. After the balance ability of patients improved, they were instructed to change from regular crutches to folding crutches. Two mineral water bottles were placed on the ground with a spacing greater than the step length to practice stepping over obstacles. Patients were instructed to step over the bottle with the affected limb. These exercises were performed in 10 daily rounds, increasing to 15 groups/day according to the rehabilitation and tolerance.

Four to eight weeks after the operation, the attending physician conducted a systematic assessment and guided the training of daily activities such as walking on the ground, walking in curves, climbing, and descending escalators. Resistance exercises of muscle groups around the hip joint were increased according to the patient’s muscle strength, and the weight-bearing of the affected limb was increased until the weight-bearing could be carried out completely without equipment. Eight to 12 weeks after the operation, independent life training exercises, such as self-folding quilts, cleaning the ground, cooking, etc., were carried out. Each stage of the training regimen was strictly monitored; door-to-door visits were conducted once a month. Alternatively, patients were instructed to return to the hospital for motor function tests and evaluations. Under the guidance of the attending doctor, housework exercises were carried out to prevent excessive weight-bearing or excessive activity from affecting functional rehabilitation.

### The following observation indexes were collected:

Hip function. Harris hip score scale (HHS) was used to evaluate the hip function of the two groups, with a total score of 100 and a higher score indicating better hip function. The functional independence measure (FIM) scale was used to evaluate the functional recovery of patients. The scale was divided into seven levels, six categories, and 18 items, and the scoring method was a 7-point system. The total score of 18 items was added; the highest score was 126, and the lowest was 18. A higher score indicated better functional independence and smaller dependence.

### Motor function:

The motor function was evaluated by the 6-minute walking test (6MWT). After a complete rest of 15 minutes, the longest one-time walking distance within six minutes was recorded. The maximal distance was 50m. The Berg Balance Scale (BBS) was used to evaluate balance ability. The scale had a total score of 56 points. A higher score indicated better balance ability. The patient’s standing-walking time (time required to stand up on standing command and return to the sitting position after 3m of normal walking.

### Quality of life (QOL) and self-care ability:

The QOL is based on the QOL-C30 scale, which includes five dimensions: body, role, emotion, social function, and cognition. Each dimension is 100 points, and the total score of this study is calculated by converting 20% of each dimension. A higher score indicates better QOL. Self-care ability was evaluated according to the Barthel Index (BI), with a total of 100 points; the higher the score, the stronger the self-care ability.

### Statistical Analysis:

The data were input into Microsoft Excel and analyzed using SPSS version 24.0 (IBM Corp, Armonk, NY, USA). According to the distribution normality evaluated by the Shapiro-Wilk test, the data of nonnormal distribution were represented by median and interquartile interval, and the Wilcoxon test was used for analysis. The number of use cases represented the counting data, and the chi-square test was used for analysis. P<0.05 was considered statistically significant.

## RESULTS

**Fig.2:**
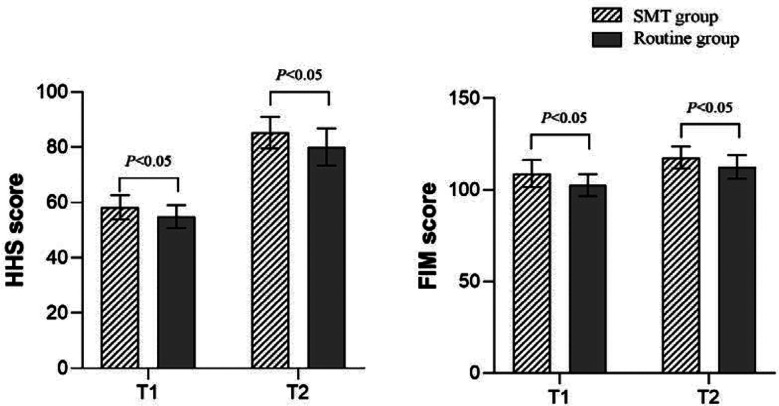
Comparison of hip joint function between the two groups after the intervention; T1: After four weeks of intervention; T2: After 12 weeks of intervention; SMT: symptom management theory; HHS: Harris hip score; FIM: function independent measurement.

**Fig.3:**
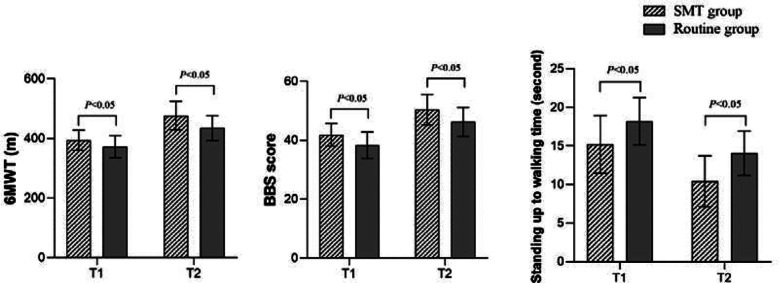
Comparison of motor function between the two groups after intervention; T1: After four weeks of intervention; T2: After 12 weeks of intervention; SMT: symptom management theory; 6WMT: 6-minute walk test; BBS: Berg balance scale.

**Fig.4:**
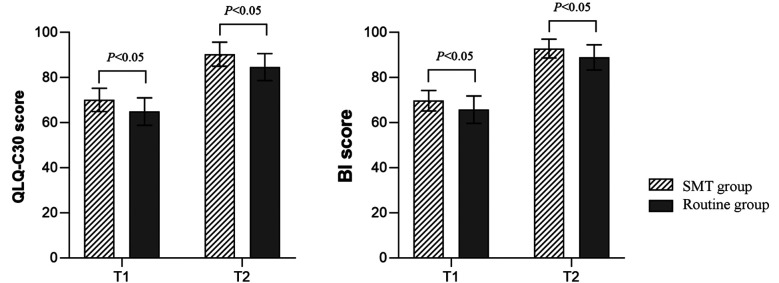
Comparison of QOL and self-care ability between the two groups; T1: After four weeks of intervention; T2: After 12 weeks of intervention; SMT: symptom management theory; QLQ-C30: Quality of Life Questionnaire Core-30; BI: Barthel Index.

There was no significant difference in clinical characteristics between the two groups (*P*>0.05) (Table-I). Fig.2 shows that after four and 12 weeks of intervention, HHS and FIM scores of the SMT group were significantly higher than those of the control group (*P*<0.05). Fig.3 shows that after four and 12 weeks of intervention, the 6MWT and BBS scores in the SMT group were significantly higher than those in the control group, while the time from standing to walking was significantly shorter than that in the control group (*P*<0.05). As shown in Fig.4, the QLQ-C30 and Bi scores of the SMT group were significantly higher compared to the control group after four and 12 weeks of intervention (*P*<0.05).

**Table-I:** Comparison of clinical characteristics between the two groups.

Characteristics	SMT group (n=60)	Control group (n=60)	Z/χ^2^	P
Age (years), M(P25/P75)	82 (77.5-83.5)	83 (76.5-86)	-1.927	0.054
Female (yes), n (%)	34 (56.7)	40 (66.7)	1.269	0.260
*Surgical reasons, n (%)*				
Femoral neck fracture	45 (75.0)	46 (76.7)	1.703	0.427
Femoral head necrosis	10 (16.7)	6 (10.0)		
Osteoarthritis of hip joint	5 (8.3)	8 (13.3)		
*Type of operation, n (%)*				
Hemiarthroplasty	28 (46.7)	30 (50.0)	0.133	0.715
THA	32 (53.3)	30 (50.0)		
*Educational level, n (%)*				
Junior high school and below	41 (68.3)	36 (60.0)	0.906	0.341
High school and above	19 (31.7)	24 (40.0)		

*Note:* SMT: symptom management theory; THA: total hip arthroplasty.

## DISCUSSION

This study showed that the staged rehabilitation guidance based on the SMT is more effective than the conventional rehabilitation regimen in improving hip function, motor function, QOL, and self-care ability of elderly patients 12 weeks after the HA surgery. In contrast to conventional rehabilitation, staged rehabilitation guidance based on SMT consists of three parts: symptom experience, symptom management strategy, and management outcome.^10^ The change in the symptom status is considered the main outcome of interest, and symptom management strategies are focused on averting, delaying, or minimizing the symptom experience. That allows for a straightforward assessment of the efficiency of intervention.^8^,^9^,^11^ Hexiaoju et al.^12^ adopted SMT-based nursing support for breast cancer patients undergoing chemotherapy and showed that this method positively impacted patients’ QOL and self-management ability. A study by Zhouxu et al.^13^ reported SMT-based nursing support in patients with colorectal cancer undergoing chemotherapy. It showed that SMT-based nursing relieved negative emotions and improved treatment compliance and QOL of patients. Although the current study focused on elderly post-HA patients, our results further confirm the benefits of rehabilitation guidance based on SMT.

This study also showed that after four and 12 weeks of intervention, the HHS and FIM scores of patients who received rehabilitation guidance based on SMT were significantly higher than those of the control group. Our results show that the staged rehabilitation guidance based on SMT can promote the recovery of hip joint function and improve self-care ability in daily life. Previous studies^14^,^15^ have shown that early muscle strength and joint range of motion training can effectively prevent and reduce joint adhesion and muscle atrophy and promote the recovery of joint function. The SMT-based staged rehabilitation guidance included early upper limb and leg training, which reduced the muscle atrophy around the hip and enhanced muscle strength. Moreover, with continuous training, patients’ activity endurance and joint flexibility gradually enhanced, further facilitating the recovery of hip joint function.

This study found that the staged rehabilitation guidance based on SMT can improve patients’ motor ability. Combining muscle strength, balance, walking training, and step-by-step exercises allows for gradual improvement of the patient’s lower limb function. From the kinematics perspective, standardized rehabilitation training is conducive to the recovery of related functions and allows patients to engage in daily household chores.^16^,^17^ Studies have shown that exercise stimulation is effective in improving patients’ physical performance (such as muscle strength and endurance) and daily activities (such as cleaning and cooking).^17^,^18^

This study used QLQ-C30 and Bi scale to evaluate the improvement of QOL after the intervention.^19^,^20^ The results showed that the staged rehabilitation guidance based on SMT positively improved the QOL and self-care ability of elderly patients. The SMT-based staged rehabilitation guidance takes into account the individual needs of elderly patients, and psychological support, which plays an important role in the process of rehabilitation, can alleviate the anxiety and depression of elderly patients and enhance their rehabilitation confidence and compliance.

In addition, this method of rehabilitation guidance includes continuous adjustment of the plan through regular evaluation and feedback mechanisms to ensure that the rehabilitation measures match the actual needs of patients. Research shows that the QOL of the elderly is affected by physiological and psychological barriers and functional limitations.^21^,^22^ In addition to postoperative functional exercise, the staged rehabilitation guidance based on SMT guides patients to carry out simple self-care tasks (such as bathing and dressing). Enhancing self-care directly promotes the independence and self-confidence of elderly patients, reduces their dependence on others, and facilitates their early return to normal social work and life.^23^

### Limitations:

The study was conducted in a single health care environment to reduce the confounding variables caused by different hospital rehabilitation programs. Moreover, this is a single-center retrospective study. Incomplete medical records and the bias of recalling medical history increase the complexity of the study and may be prone to selection bias. Additionally, the study did not distinguish between total HA and hemiarthroplasty patients. This may increase individual differences and reduce the accuracy of outcome estimation because the trajectory of postoperative recovery may differ between the two surgeries. Since the sample size of each operation is small, the results of subgroup analysis may not be conclusive.

Although there was no significant difference in clinical characteristics between the two groups, the baseline levels of hip function, motor function, QOL, and self-care ability could not be evaluated because the intervention measures were implemented on the first day after the operation, which may lead to a certain bias in the results after 12 weeks of intervention. Finally, the study data was only recorded after 12 weeks of intervention, and the long-term impact of staged rehabilitation guidance based on SMT is unclear.

## CONCLUSION

Compared with conventional rehabilitation, staged rehabilitation guidance based on SMT can improve hip joint and motor function, QOL, and self-care ability of elderly patients after HA.

### Authors’ Contributions:

**YX:** Study design, literature search and manuscript writing.

**YX and LC:** Data collection, data analysis, interpretation and critical review.

**YX:** Manuscript revision and validation, Critical analysis.

All authors have read, approved the final manuscript and are responsible for the integrity of the study.
